# σ^54^-dependent regulome in *Desulfovibrio vulgaris* Hildenborough

**DOI:** 10.1186/s12864-015-2176-y

**Published:** 2015-11-10

**Authors:** Alexey E. Kazakov, Lara Rajeev, Amy Chen, Eric G. Luning, Inna Dubchak, Aindrila Mukhopadhyay, Pavel S. Novichkov

**Affiliations:** Lawrence Berkeley National Laboratory, Berkeley, CA 94710 USA; Department of Energy Joint Genome Institute, 2800 Mitchell Drive, Walnut Creek, CA 94598 USA

**Keywords:** Transcription factor, Transcriptional regulation, Sigma factor, *Desulfovibrio vulgaris*, Enhancer binding proteins

## Abstract

**Background:**

The σ^54^ subunit controls a unique class of promoters in bacteria. Such promoters, without exception, require enhancer binding proteins (EBPs) for transcription initiation. *Desulfovibrio vulgaris* Hildenborough, a model bacterium for sulfate reduction studies, has a high number of EBPs, more than most sequenced bacteria. The cellular processes regulated by many of these EBPs remain unknown.

**Results:**

To characterize the σ^54^-dependent regulome of *D. vulgaris* Hildenborough, we identified EBP binding motifs and regulated genes by a combination of computational and experimental techniques. These predictions were supported by our reconstruction of σ^54^-dependent promoters by comparative genomics. We reassessed and refined the results of earlier studies on regulation in *D. vulgaris* Hildenborough and consolidated them with our new findings. It allowed us to reconstruct the σ^54^ regulome in *D. vulgaris* Hildenborough. This regulome includes 36 regulons that consist of 201 coding genes and 4 non-coding RNAs, and is involved in nitrogen, carbon and energy metabolism, regulation, transmembrane transport and various extracellular functions. To the best of our knowledge, this is the first report of direct regulation of alanine dehydrogenase, pyruvate metabolism genes and type III secretion system by σ^54^-dependent regulators.

**Conclusions:**

The σ^54^-dependent regulome is an important component of transcriptional regulatory network in *D. vulgaris* Hildenborough and related free-living *Deltaproteobacteria*. Our study provides a representative collection of σ^54^-dependent regulons that can be used for regulation prediction in *Deltaproteobacteria* and other taxa.

**Electronic supplementary material:**

The online version of this article (doi:10.1186/s12864-015-2176-y) contains supplementary material, which is available to authorized users.

## Background

Sigma subunits of bacterial RNA polymerase regulate the process of transcriptional initiation through binding to RNA polymerase holoenzyme, recognition of promoter sequences, and promotion of open complex formation. One of the sigma factors, called alternative sigma factor σ^54^, significantly differs from all other known sigma factor proteins in two respects (reviewed in [1]). First, it recognizes and binds characteristic promoters with GG −24 and TGC −12 elements that are more conserved than the promoters of σ^70^ family. Second, σ^54^ strictly requires the ATP hydrolysis activity of an activating transcription factor (TF) for the formation of open promoter complex. TFs necessary for such activation are called prokaryotic enhancer-binding proteins (EBPs) [2]. EBPs specifically bind a conserved upstream activating sequence (UAS) that contains one or several TF binding sites. UASs are located 100 bp or more upstream from the σ^54^-dependent promoter, and the interaction of σ^54^-polymerase complex with EBP oligomer requires DNA looping between the UAS and promoter. This looping is often facilitated by bacterial chromatin proteins like integration host factor or histone-like protein HU. Thus, initiation of σ^54^-dependent transcription involves promoter binding by σ^54^-containing RNA polymerase, UAS binding by EBP, and activation of σ^54^-polymerase by ATP hydrolysis with subsequent formation of an open complex.

The analysis of taxonomic diversity of *rpoN* (the gene encoding σ^54^) and EBP-encoding genes demonstrated their presence in about 60 % of bacterial genomes [3]. σ^54^ seems to be an essential protein in some *Deltaproteobacteria*, since attempts to produce viable *rpoN* deletion mutants of *Myxococcus xanthus* [4] and *Geobacter sulfurreducens* [5] were unsuccessful.

σ^54^-dependent regulons (called sigmulons) have been extensively studied in several model organisms. It was found that in *Escherichia coli*, the σ^54^ sigmulon includes about 30 operons (reviewed in [6]), and 14 of them are involved in nitrogen metabolism. The products of other σ^54^-dependent operons contribute to formate, propionate and acetolactate metabolism, zinc tolerance, phage shock response and other functions. Most of *E. coli* EBPs control one or few operons, and only NtrC regulon includes 14 operons. However, in other model organisms EBPs may control a larger number of promoters. For example, NifA in *Rhizobium etli* regulates 43 operons involved in nitrogen fixation, energy production, transport, secondary metabolism and other diverse functions [7]. In *Pseudomonas putida*, there are 22 EBP-encoding genes and most of them are located close to σ^54^-dependent operons [8]. Of 46 σ^54^-dependent promoters in *P. putida*, 36 are related to nitrogen and carbon metabolism, flagella and motility. A much larger σ^54^-dependent sigmulon (108 promoters) was described in *Geobacter sulfurreducens* [5]. In this bacterium, σ^54^-dependent genes are involved in nitrogen assimilation, formate and amino acid metabolism, C_4_-dicarboxylate transport, flagella and pili biogenesis and other functions. Nevertheless, some bacteria have very small σ^54^ sigmulons. For example, *Lactobacillus plantarum* has only one EBP that activates a single σ^54^-dependent promoter upstream of mannose transport operon [9].

**Fig. 1:**
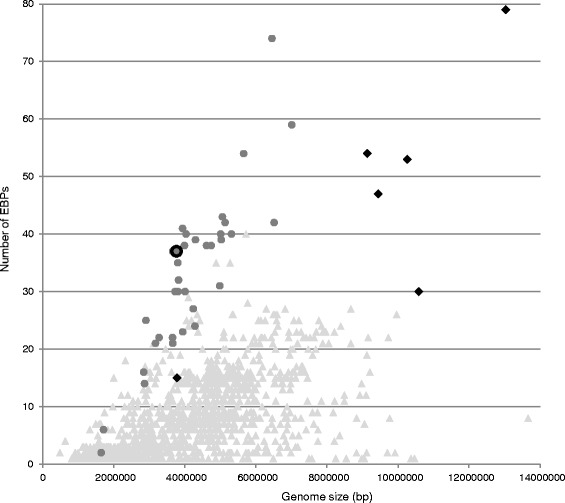
The number of EBPs in the bacterial genomes of different sizes. Grey circles represent anaerobic *Deltaproteobacteria*, and black diamonds represent aerobic *Deltaproteobacteria. D. vulgaris* Hildenborough marked by a black ring. Grey triangles represent other bacterial genomes

As σ^54^-dependent transcription requires EBP for initiation, the range of functions regulated by σ^54^ directly depends on the assortment of EBPs in the bacterium. A search within the MicrobesOnline database demonstrates the presence of EBPs in 1435 bacterial species [10]. Aerobic *Deltaproteobacteria* with large genomes have the highest total number of EBPs (Fig. 1, Additional file 1). A high number of EBPs in *Myxococcus xanthus* and related bacteria can be explained not only by their genome size, but also by wide involvement of these regulators in the very complex fruiting body development program [11]. However, the highest relative number of EBPs (with relation to genome size) was observed in anaerobic *Deltaproteobacteria*, isolated from soil and aquatic habitats. *Desulfovibrio vulgaris* strains are in the third place on the list after *Desulfomonile tiedjei* and *Desulfomicrobium baculatum.*

One of these strains*, Desulfovibrio vulgaris* Hildenborough, a model bacterium for sulfate reduction studies, has 37 genes encoding putative σ^54^-associated EBPs [12]. Among these, both target genes and TF binding motifs are known for 14 EBPs [13], and only the target genes for 6 more EBPs [13, 14]. Putative σ^54^-dependent promoters were predicted computationally upstream of many EBP target operons [13, 14]. A genome-wide analysis of *D. vulgaris* Hildenborough transcriptome [15] has identified 70 σ^54^-dependent promoters by a combination of 5′-RNA-seq and computational analysis of promoter motifs.

Despite the existing data on σ^54^-dependent transcription in *D. vulgaris* Hildenborough, we still do not have a complete understanding of the role of the σ^54^-dependent regulome (defined as the compendium of regulatory elements that facilitate σ^54^-dependent transcription) in this bacterium. For most of σ^54^-dependent promoters, we do not know EBPs that activate them. We also do not know binding motifs and target genes for multiple EBPs. Thus, the goal of the current study was to comprehensively reconstruct the σ^54^-dependent regulome in *D. vulgaris* Hildenborough genome-wide. We inferred several σ^54^-dependent regulons not known before (including those associated with type III secretion, electron transport, pyruvate and formate metabolism). This allowed us to make important conclusions about the set of functions controlled by σ^54^-associated regulators in *D. vulgaris* Hildenborough and related *Deltaproteobacteria*.

## Results and discussion

In order to characterize a particular type of transcriptional regulation in bacteria, one needs to elucidate all the necessary components in this process, which typically are: transcriptional regulator, its binding sites, and a set of regulated genes or operons. In our study we aimed at discovering the compendium of regulatory elements that facilitate σ^54^-dependent transcription, i.e., σ^54^-dependent regulome. This regulome consists of all σ^54^-dependent transcriptional activators (EBPs), EBP binding sites and EBP-regulated operons with σ^54^-dependent promoters. We started with the computational analysis of all predicted *D. vulgaris* Hildenborough EBPs to verify the presence of DNA-binding domain and σ^54^-interacting domain. Next, we reconstructed EBP binding motifs (if unknown) and identified EBP binding sites in the *D. vulgaris* Hildenborough genome. Finally, we compared EBP regulons with the σ^54^-dependent sigmulon and identified a set of operons co-regulated by EBPs and σ^54^ subunit.

### The repertoire of σ^54^-dependent regulators in *D. vulgaris* Hildenborough

All σ^54^-dependent EBPs possess a central AAA+ domain responsible for ATP binding and hydrolysis, oligomerization and interaction with σ^54^ subunit [2]. A common feature of σ^54^-dependent EBPs is the peptide motif ‘GAFTGA’, a structural element of AAA+ domain that interacts with sigma subunit protein [2]. We checked the presence of this motif in 37 EBPs from *D. vulgaris* Hildenborough by comparing them with AAA+ domain model from PFAM database (described in Methods). An exact GAFTGA sequence was observed in 25 of 37 EBPs, while in the other 12 EBPs this motif has different substitutions (Table 1). Ten of these 12 EBPs possess eight variations of the motif (GAFSEA, GAFSGA, GAFTDA, GAFTGG, GAFTHA, GAYTDA, GAYTGS, GSFTGA) that would maintain the EBP’s ability to activate transcription [2]. The remaining two EBPs (DVUA0100 and DVU2960) have substitutions in the GAFTGA motif that might interfere with its ability to bind σ^54^ protein. DVUA0100 possesses three substitutions (GVATGV), but our regulon prediction results (see below) suggest that they do not prevent DVUA0100 from activating σ^54^-dependent promoters. The second protein, DVU2960, has only one substitution of highly conserved threonine for proline (GAFPGA). Although an earlier work [3] reported a single amino acid deletion in the GAFPGA motif of DVU2960, our comparison with the AAA+ domain model does not confirm the deletion. Nevertheless, the functionality of DVU2960 as a σ^54^-dependent activator is unclear, because the same substitution of threonine for proline in another σ^54^-dependent activator, NifA from *Bradyrhizobium japonicum*, rendered the protein inactive [2].

**Table 1:** Enhancer binding proteins of *D. vulgaris* Hildenborough

Regulator	Type	GAFTGA motif	Target operons	Function	Regulon reference
DVU0110	TCS response regulator	GAFTGA	DVU0132-DVU0133, DVU1152	Exopolysaccharide and biofilm synthesis,transmembrane transport	Rajeev et al., 2011 [13], this study
DVU0118	TCS response regulator	GAFTGA	DVU0123-DVU0121	Transmembrane transport	Rajeev et al., 2011 [13]
DVU0151	Single-component regulator	GAFSEA	DVU0150-DVU0146	Transmembrane transport	This study
DVU0539	TCS response regulator	GAFTGA	DVU0542-DVU0545, DVU0943-DVU0946, DVU2133-DVU2132, DVU3025-DVU3033	Lactate metabolism, transmembrane transport	Rajeev et al., 2011 [13]
DVU0569	Single-component regulator	GAFTGG	DVU0571	Amino acid metabolism	This study
DVU0619	TCS response regulator	GAFTGA	DVU0617-DVU0616		This study
DVU0621	TCS response regulator	GAFTGA	DVU0624-DVU0625, DVU3025-DVU3033	Nitrite stress response, lactate metabolism	Rajeev et al., 2011 [13], this study
DVU0653	TCS response regulator	GAFTGA	ncRNA downstream of DVU0653	Posttranscriptional regulation	Rajeev et al., 2011 [13], this study
DVU0679	TCS response regulator	GAYTGS	ncRNA downstream of DVU0679	Posttranscriptional regulation	Rajeev et al., 2011 [13]
DVU0744	TCS response regulator	GAFTGA	DVU0682	Transcriptional regulation	Rajeev et al., 2011 [13], this study
DVU0804	TCS response regulator	GAFTGA	DVU0805-DVU0806	Posttranscriptional regulation	Rajeev et al., 2011 [13], this study
DVU0946	TCS response regulator	GAFTGA	DVU0542-DVU0545, DVU0943-DVU0946, DVU2133-DVU2132	Lactate metabolism, transmembrane transport	Rajeev et al., 2011 [13]
DVU1063	TCS response regulator	GAFTGA	DVU0307, DVU0316-DVU0310, DVU0318, DVU0320, DVU0863-DVU0862, DVU1032, DVU1441, DVU1444, DVU1445-DVU1443, DVU1805, DVU1880, DVU2090	Flagella	Rajeev et al., 2011 [13], this study
DVU1156	TCS response regulator	GAFTGA	DVU1164	Amide metabolism	Rajeev et al., 2011 [13], this study
DVU1419	TCS response regulator	GAFTGA	DVU0036, DVU1418-DVU1419, ncRNA upstream of DVU3282	Envelope stress response, posttranscriptional regulation	Rajeev et al., 2011 [13]
DVU1949	Single-component regulator	GAFTGA	DVU1231-DVU1233, DVU1258, DVU3392*, DVU1952-DVU1949*, DVU0671*, DVU3290-DVU3292*, DVU2343-DVU2340*, DVU1823-DVU1821*, DVU0753-DVU0751*	Nitrogen metabolism and transport	This study
DVU2106	Single-component regulator	GAFTDA	DVU2105-DVU2103, DVU2107-DVU2109	Cell division	Fievet et al., 2011
DVU2114	TCS response regulator	GAFTGA	DVU3342-DVU3344, DORF39640-DVU2129	Pili	Rajeev et al., 2011 [13]
DVU2275	Single-component regulator	GAYTDA	DVU2272-DVU2269	Pyruvate metabolism and transport	This study
DVU2359	Single-component regulator	GAFTGA	ncRNA upstream of DVU2357	Posttranscriptional regulation	This study
DVU2394	TCS response regulator	GSFTGA	DVU2405-DVU2399	Energy metabolism	Rajeev et al., 2011 [13]
DVU2827	Single-component regulator	GAFSGA	DVU2820, DVU2822-DVU2825	Pyruvate metabolism and transport	This study
DVU2894	Single-component regulator	GAFTHA	DVU0047-DVU0043, DVU0410-DVU0409, DVU2894*	Flagella	This study
DVU2934	TCS response regulator	GAFTGA	DVU2917	Lipid A biosynthesis	Rajeev et al., 2011 [13]
DVU2956	Single-component regulator	GAFTGA	DVU2957-DVU2964	Transmembrane transport	This study
DVU2960	Single-component regulator	GAFPGA			
DVU2989	Single-component regulator	GAFTGA	DVU2988-DVU2986	Phage shock response	This study
DVU3023	TCS response regulator	GAFTGA	DVU2451, DVU3025-DVU3033, DVU3284*	Lactate metabolism and transport	Rajeev et al., 2011 [13]
DVU3142	Single-component regulator	GAFTGA	DVU3143-DVU3145	Energy metabolism	This study
DVU3220	TCS response regulator	GSFTGA	DVU1231-DVU1233	Nitrogen metabolism and transport	Rajeev et al., 2011 [13]
DVU3305	TCS response regulator	GAFSGA	DVU3302-DVU3298, DVU3303-DVU3305*	General stress response, transmembrane transport	Rajeev et al., 2011 [13]
DVU3334	TCS response regulator	GAFTGA	DVU3339.1-DVU3337	Potassium uptake	Rajeev et al., 2011 [13]
DVU3381	TCS response regulator	GAFTGA	DVU3382-DVU3381, DVU3384	Envelope stress response	Rajeev et al., 2011 [13]
DVUA0024	TCS response regulator (pseudogene)	GAFTGA			
DVUA0057	TCS response regulator	GAFTGA	DVU0132-DVU0133, DVU1152, DVUA0030, DVUA0032-DVUA0031, DVUA0089, DVUA0036-DVUA0041	Exopolysaccharide and biofilm synthesis	Rajeev et al., 2011 [13], this study
DVUA0100	Single-component regulator	GVATGV	DVU4010, DVUA0106-DVUA0099, DVUA203-DVUA0125	Type III secretion	This study
DVUA0143	Single-component regulator	GAFTGA	DVUA0015-DVUA0007, DVUA0016	Nitrogen metabolism	This study

Operons that do not have σ^54^-dependent promoters are marked with asterisk

**Fig. 2:**
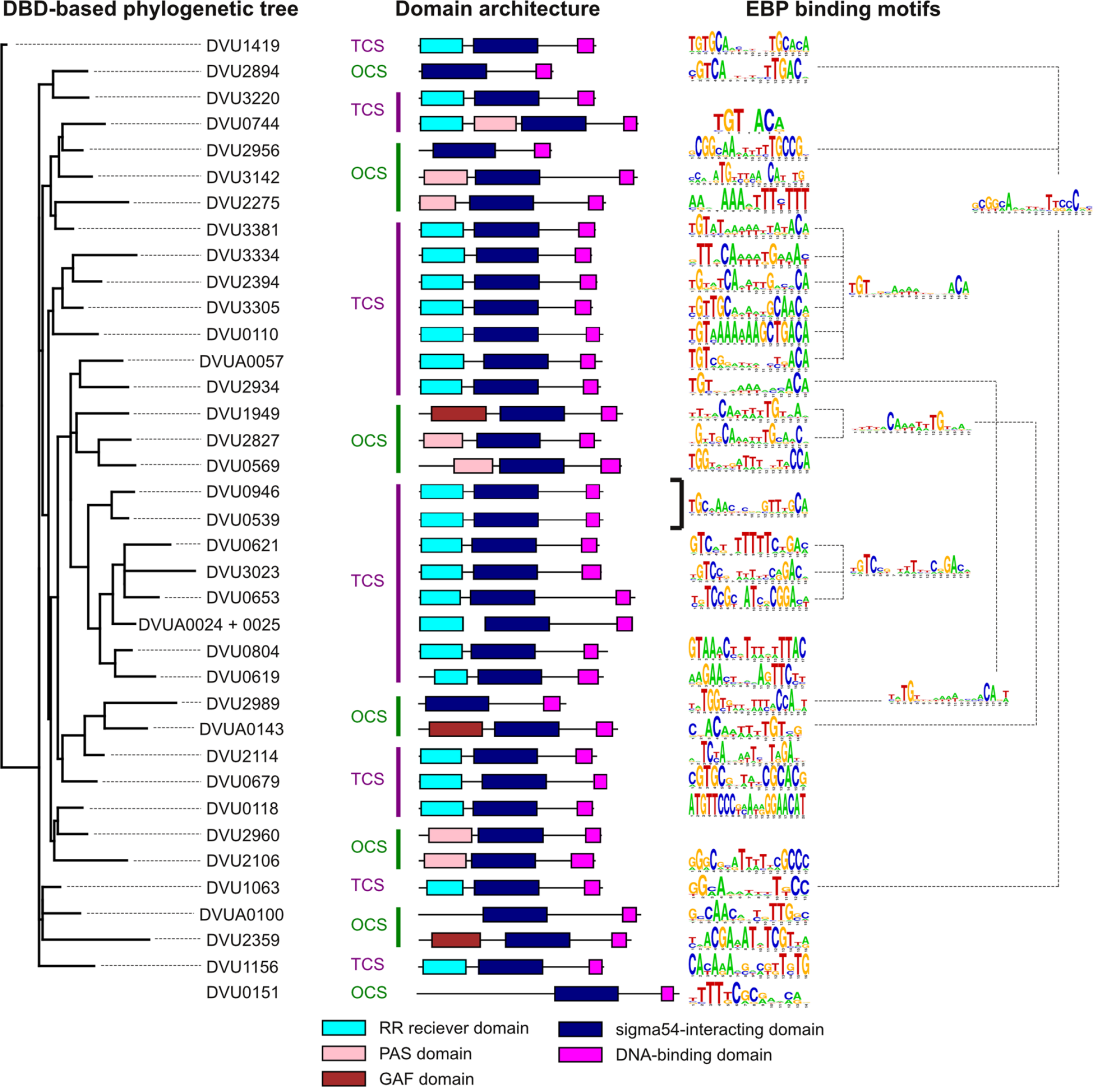
Phylogenetic tree of *D. vulgaris* Hildenborough EBPs based on DNA-binding domains. DVU0151 protein was omitted from this tree because it has a different DNA-binding domain sequence. Domain architecture and TF binding motifs are shown for each protein, where available. DVU0946 and DVU0539 EBPs share a common binding motif (indicated by bracket). Groups of similar motifs identified by TOMTOM comparison are displayed

Many known EBPs have one or two N-terminal domains that modulate transcriptional activation capability of the regulator [2]. These domains recognize various environmental signals through phosphorylation or ligand binding. In particular, 22 EBPs of *D. vulgaris* Hildenborough are response regulators of two-component systems (TCS) with a N-terminal response regulator receiver domain (Fig. 2) that mediates phosphotransfer from a sensor histidine kinase. The other 15 EBPs are one-component regulatory systems (OCS) containing both input and output domains in a single protein, and lacking histidine kinase and response regulator domains. The two most common sensing domains of OCSs are GAF (3 proteins) [16] and PAS domains (6 proteins) [17]. One of the response regulator proteins, DVU0774, also has a PAS domain, which is suggestive of signal integration or dual activation. Besides PAS and GAF domains, we observed two OCSs with long conserved N-terminal regions that may encompass unknown sensing domains (DVU0151 and DVUA0100). Finally, 4 out of the 37 EBPs (DVU2894, DVU2956, DVU2989 and DVUA0024) lack N-terminal domains suggesting either an interaction with a partner protein that affects the regulatory activity of the EBP, or participation of these proteins in a regulatory cascade. Such interactions have been described in other species, for example, PspA protein negatively regulating PspF transcription factor [18].

The vast majority of known EBPs have a DNA-binding domain that is located at the C-terminus of the protein [2]. In all *D. vulgaris* Hildenborough EBPs but one, there are annotated Fis-type HTH motifs (Additional file 2), which are typical for σ^54^-dependent regulators [19]. The only exception is DVU0151 that does not have an annotated C-terminal DNA-binding domain and thus is not included in the phylogenetic tree constructed by DNA-binding domains (Fig. 2).

Our examination of DVU0151 and its orthologs from other *Desulfovibrionales* revealed that some of them (DvMF_2340, Dde_0697 and Dbac_2039) have matches to the winged helix DNA-binding domain model (SSF46785) from the SUPERFAMILY database [20], thus it is likely that DVU0151 orthologs have DNA-binding activity. Multiple sequence alignment of these proteins demonstrates the presence of strong sequence conservation at the location of the SSF46785 domain across all proteins, including DVU0151 (Additional file 3). This similarity further suggests that DVU0151 is a DNA-binding regulatory protein indeed. Thus, we predict that all 37 σ^54^-dependent regulators from *D. vulgaris* Hildenborough bind DNA in a sequence-specific manner, similar to a vast majority of other bacterial EBPs [2].

### Reconstructed TF binding motifs for σ^54^-dependent regulators in *D. vulgaris* Hildenborough

**Fig. 3:**
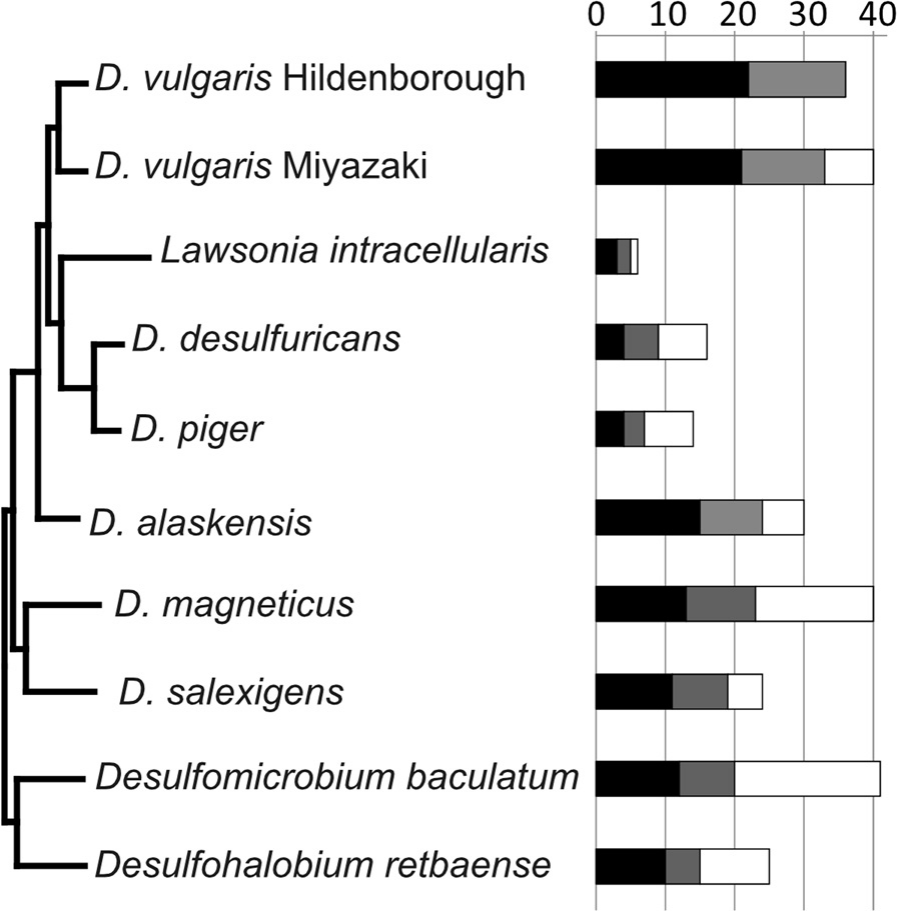
Phylogeny of *Desulfovibrionales* and conservation of *D. vulgaris* Hildenborough EBPs in closely related genomes. One-component EBPs from *D. vulgaris* Hildenborough and their orthologs are marked grey, σ^54^-dependent response regulators of TCSs from *D. vulgaris* Hildenborough and their orthologs are marked black, other EBPs (that lack orthologs in *D. vulgaris* Hildenborough) are marked white. Phylogenetic tree of *Desulfovibrionales* was extracted from the MicrobesOnline species tree [10], EBP orthologs were calculated as described in Methods section

In order to find the genes and operons that compose the regulon of each EBP, we reconstructed TF binding motifs for all these regulators and used those motifs in comparative genomics analysis. As the first step in motif reconstruction, we identified orthologs of all 37 *D. vulgaris* Hildenborough EBPs in nine species of *Desulfovibrionales*, as described in Methods. The number of EBPs in these genomes varies significantly, from 6 in *Lawsonia intracellularis* to 41 in *Desulfomicrobium baculatum* (Fig. 3). As expected, *D. vulgaris* Miyazaki, the closest relative of *D. vulgaris* Hildenborough, has 33 EBPs conserved between these two species, the highest number among the studied genomes*.* A little more distant *L. intracellularis*, *D. desulfuricans* and *D. piger* (see phylogenetic tree on Fig. 3) have strikingly lower numbers of EBP orthologs (5–9), due to a lower number of EBPs in these genomes in general. The number of EBP orthologs in the more distant *Desulfovibrionales* species gradually decrease from 24 in *Desulfovibrio alaskensis* G20 to 15 in *Desulfohalobium retbaensae*, with increase of phylogenetic distance from *D. vulgaris* Hildenborough (Fig. 3). There is no significant difference between conservation of TCS response regulators and that of one-component EBPs.

It is interesting that DVUA0024 protein lacks any N-terminal signal domain but all its orthologs have response regulator receiver domain. We determined that DVUA0025 protein, encoded by a gene immediately upstream of DVUA0024*,* is an ortholog of this domain. In addition, DVUA0024 protein lacks eight N-terminal amino acids in the AAA+ domain interacting with σ^54^ subunit. By comparing the nucleotide sequences of the closely related *D. vulgaris* strains Hildenborough, DP4 and RCH1, we identified a single-nucleotide deletion in the genome of Hildenborough strain (data not shown). This deletion leads to the premature termination of DVUA0025 protein synthesis within the σ^54^-interacting domain. As *D. vulgaris* Hildenborough is unable to synthesize the complete functional protein, we excluded DVUA0024 from subsequent regulon analysis. Nevertheless, it is possible that this protein retains some transcriptional activity*.*

**Fig. 4:**
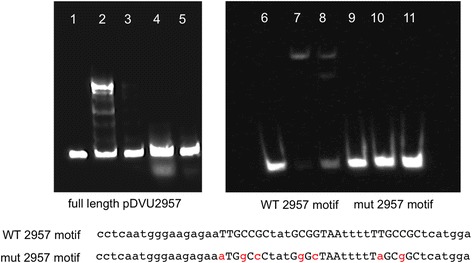
Transcriptional regulator DVU2956, and not DVU2960, binds upstream of target gene DVU2957. Lanes 1–5 show electrophoretic mobility shift assays with full-length (370 bp) upstream region of DVU2957: lane 1 – DNA only; lanes 2 and 3 – with purified DVU2956 protein (260 and 52 pmol, respectively); lanes 4 and 5 – with purified DVU2960 protein (400 and 80 pmol, respectively). Lanes 6–11 show EMSAs with predicted binding site motif upstream of DVU2957 (wild-type motif –lanes 6–8; mutated motif – lanes 9–11). Sequences of the two sites are shown below the gel image; the three conserved half-sites are shown in caps, and substitutions made in the mutated site are shown in red. Lanes 6, 9 – DNA only; lanes 7, 10 – with 390 pmol of DVU2956 protein; lanes 8, 11 – with 260 pmol of DVU2956 protein

Our analysis of EBP conservation in *Desulfovibrionales* demonstrated a good potential for comparative reconstruction of regulons. Previous studies of EBPs in *D. vulgaris* Hildenborough [13, 14] identified binding sites for 14 EBPs. In this work, we reconstructed TF binding motifs for 19 other EBPs (Fig. 2, see Methods for details). Briefly, we used comparative reconstruction of σ^54^-dependent promoters in combination with regulator conservation results and existing DAP-chip data for several regulators [13]. In many cases, we found EBP-encoding genes to be co-localized with the regulated σ^54^-dependent promoters, and this co-localization was conserved within the *Desulfovibrionales* order. In most cases, such co-localization allowed the identification of a single regulatory gene. Only in one case, two regulatory genes, DVU2956 and DVU2960, are co-localized with the same σ^54^-dependent promoter in several genomes. Using purified proteins, we tested both candidate regulators in vitro for their ability to bind predicted sites, and found that only DVU2956 shows binding (Fig. 4).

All the identified TF binding motifs were compared by TOMTOM [21], since similarity of motifs could suggest a cross-regulation between different EBPs. All-against-all comparison of 33 EBP binding motifs found similarity in only 17 motifs that could be grouped into five based on similarity (Fig. 2). In three of these groups, motifs consist of half sites TGT and ACA separated by 9, 11 or 12 bp. It is interesting that similarity of TF binding motifs do not always reflect similarity of DNA-binding domains, as demonstrated by a comparison of the groups of motifs with a phylogenetic tree based on DNA-binding domains (Fig. 2). Nearly half of the motifs (16 out of 33) did not show similarity to any other motif.

**Fig. 5:**
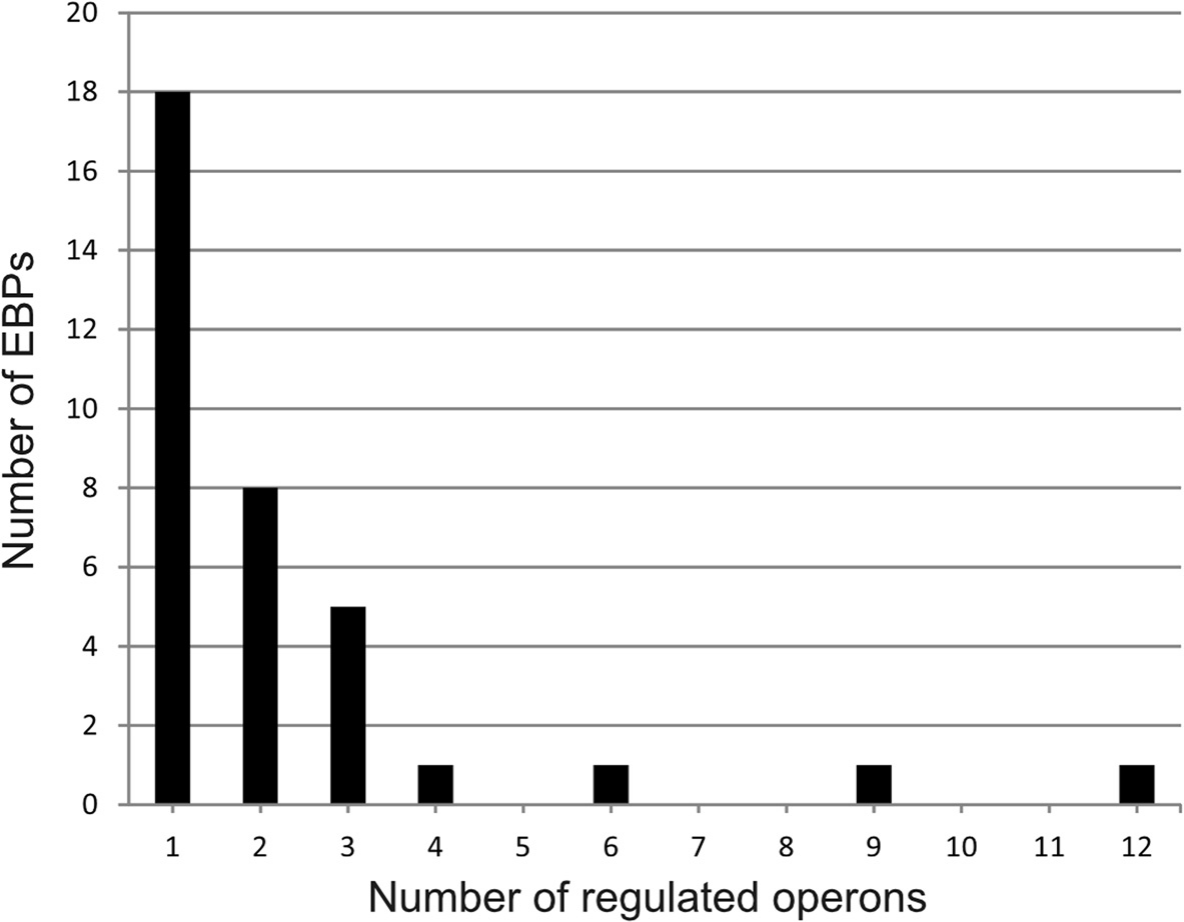
Distribution of sizes of EBP regulons

Using the collection of these EBP binding motifs, we reconstructed or confirmed regulons of 34 EBPs by a whole-genome comparative analysis (Additional file 4). For one additional EBP, DVU3220, we had no predicted motif, thus its regulated genes were deduced from experimental results [13]. For the majority of σ^54^-dependent regulators, the regulons consist of just one to four regulated operons (Fig. 5). Only DVUA0057, DVU1949 and DVU1063 regulators have larger regulons with 6, 9 and 12 operons, respectively.

### Functional diversity of σ^54^-dependent genes in *D. vulgaris* Hildenborough

We identified the content of σ^54^ sigmulon and 33 EBP regulons in *D. vulgaris* Hildenborough by a combination of computational and experimental data analysis (see Methods). We found 87 σ^54^-dependent promoters upstream of 85 operons that encompass 270 genes from different functional groups (Table 2, Additional files 5 and 6). The largest functional group (37 operons) includes genes involved in flagellar assembly and other functions linked to cell exterior: appendages, cell wall, secreted exopolysaccharides and biofilm components. This is in agreement with results of a recent large-scale analysis that demonstrated a connection of σ^54^-dependent transcription to the make-up of the bacterial exterior [3].

**Table 2:** Summary of functions regulated by σ^54^-dependent operons. Functional assignment of individual operons shown in Additional file 5

Category	Function	Operons	Regulators
Motility and cell exterior	Flagella	19	2
	Chemotaxis	2	
	Pili	3	1
	Type III secretion	4	1
	Exopolysaccharide and biofilm synthesis	7	2
	Cell wall	1	
	Lipid A biosynthesis	1	1
Metabolism and transport	Nitrogen metabolism and transport	4	3
	Amino acid metabolism and transport	2	1
	Pyruvate metabolism and transport	3	2
	Lactate metabolism and transport	2	4
	Amide metabolism	1	1
	Energy metabolism	2	2
	Potassium uptake	1	1
	Other transmembrane transport	9	6
Regulation	Transcriptional regulation	4	3
	Posttranscriptional regulation	6	5
Stress response	Envelope stress response	2	2
	Nitrite stress response	1	1
	Phage shock response	1	1
Other functions	Cell division	2	1
	Function unknown	8	1

Another large group of operons from σ^54^ sigmulon (24 operons) is comprised of metabolic and transport genes. A significant part of this group consists of genes of ammonia assimilation, nitrogen fixation and catabolism of amino acids and amides. Other genes in this group are involved in the metabolism of carboxylic acids, which are important carbon and energy sources for *D. vulgaris* and related species. σ^54^-dependent regulation of nitrogen metabolism, as well as the regulation of energy production, has been described in various taxonomic groups of bacteria (reviewed in [2]). However, σ^54^-dependent transcription of pyruvate and lactate metabolism genes have not been described in any other taxa. Nine operons in this group encode transporters from TSUP (toluene sulfonate uptake permease [22]) or PSE (putative sulfate export [23]) families. There is no data on substrate specificity of these transporting systems, but two of the PSE family transporters have been shown to be co-regulated with lactate metabolism genes [13].

Of the remaining 24 operons in σ^54^ sigmulon, 9 are involved in regulation, 5 in stress response, 2 in cell division and 8 have no assigned function. Regulatory RNAs and coding genes with σ^54^-dependent promoters are involved in transcriptional and posttranscriptional regulation, thus suggesting an existence of regulatory cascades possibly integrating different extracellular and/or intracellular signals. For some of the functions listed, transcriptional control by σ^54^ and EBPs has been shown in *Deltaproteobacteria* and other taxonomic groups (reviewed in [2]). The most studied examples of such functions are flagellar assembly and nitrogen metabolism. Since the homologous EBPs involved in the response to metal stress and phage shock have been documented in very distant bacteria such as *E. coli* [24], *Salmonella enterica* [2] and *Yersinia enterocolitica* [25], the conservation of these functions in σ^54^–dependent regulome of *D. vulgaris* Hildenborough emphasizes importance of such systems for bacteria.

In addition to the functions that are commonly regulated by σ^54^ and EBPs, we observed several EBP regulons that have not been described in any other bacteria. The four most interesting cases of σ^54^-dependent regulators for type III secretion, pyruvate transport metabolism, electron transfer and alanine dehydrogenase are described in detail below.

#### Regulator of type III secretion genes (DVUA0100)

The type III secretion system (T3SS) is a large multisubunit complex secreting effector proteins across bacterial membranes [26]. In *D. vulgaris* Hildenborough, T3SS components are encoded by two divergent operons with σ^54^-dependent promoters on the native pDV1 plasmid [27], and the gene encoding DVUA0100 EBP that is located in one of these operons. We predicted a putative DVUA0100 binding motif in the common upstream region of these two operons in three *Desulfovibrio* spp. genomes (Fig. 2). A whole-genome regulon analysis identified additional regulatory sites upstream of DVU4010 gene and its orthologs in two other genomes. These genes have predicted σ^54^-dependent promoters, and their products are annotated as hypothetical proteins. Using a computational tool for prediction of effector proteins of type III secretion system [28], we demonstrated a high probability for DVU4010 to be a secreted T3SS effector (NaiveBayes Prediction Score = 0.994). Colocalization of DVU4010 gene with DVU2392 gene encoding type III secretion chaperone on the chromosome supports our functional assignment.

#### Regulators of pyruvate formate-lyases (DVU2275 and DVU2827)

**Fig. 6:**
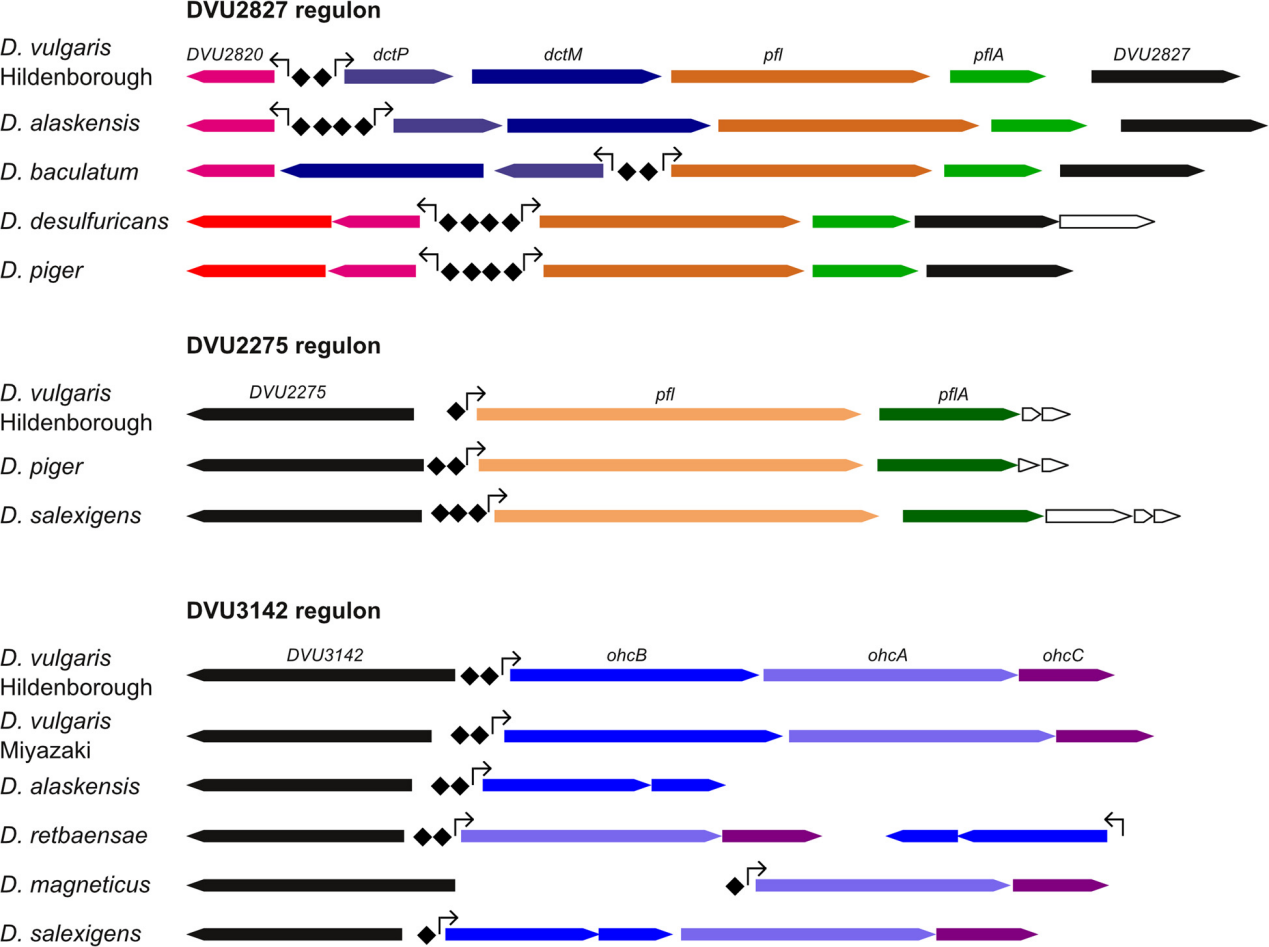
DVU2827, DVU2275, DVU3142 regulons and their conservation in other *Desulfovibrionales*. EBP-encoding genes are marked black, promoters are shown by arrows, EBP binding sites are shown by diamonds. Genes encoding MFS transporters are marked red, genes encoding amydohydrolase family proteins are marked magenta, genes encoding PFL are marked orange, genes encoding PFL-activating enzymes are marked green

Pyruvate formate-lyase (PFL) catalyzes the reversible nonoxidative dissimilation of pyruvate to acetyl-coenzyme A and formate [29]. *D. vulgaris* Hildenborough genome contains two genes encoding PFLs, DVU2272 and DVU2824 [30], and both of them are co-transcribed with genes encoding PFL-activating proteins. DVU2824-encoding operon also contains genes for C4-dicarboxylate transporting system, DVU2822 and DVU2823 (Fig. 6). We observed that orthologs of two EBP-encoding genes, DVU2275 and DVU2827, are co-localized with PFL operons in *Desulfovibrionales* genomes, and these operons had conserved σ^54^-dependent promoters. We predicted that DVU2275 regulator binds an AT-rich palindromic site upstream of the DVU2272 promoter (Fig. 6). DVU2271 gene demonstrated increased expression during growth on hydrogen and sulfate [31], thus suggesting that the DVU2272-DVU2269 operon may be involved in pyruvate biosynthesis when acetate and CO_2_ are the only carbon sources available.

We also predicted DVU2827 binding sites upstream of operons encoding pyruvate-formate lyase DVU2824 and its orthologs in five *Desulfovibrionales* genomes (Fig. 6). These sites probably activate a neighbor gene DVU2820 encoding amidohydrolase family protein, which also has σ^54^-dependent promoter. Orthologs of DVU2820 in *D. desulfuricans* and *D. piger* (Ddes_11438 and DESPIG_02846) are co-transcribed with genes encoding major facilitator superfamily (MFS) transporters. These two genomes lack C4-dicarboxylate transporters encoded by DVU2822 and DVU2823 orthologs. Search in Transporter Classification Database [PMID: 24225317] identified similar MFS proteins from metabolite:H+ symporter family (2.A.1.6) that can transport citrate, oxoglutarate, dicarboxylates, acetate and shikimate (e-value below e^−46^). These data suggest that MFS transporters Ddes_1438 and DESPIG_02846 may transport C4-dicarboxylates.

#### Regulator of electron transport system (DVU3142)

It was previously demonstrated that octaheme cytochrome complex (Ohc) transports electrons from a periplasmic octahemic c-type cytochrome OhcA to an iron-sulfur membrane protein OhcB and then to a haem *b*-containing membrane protein OhcC [32]. In *D. vulgaris* Hildenborough Ohc complex proteins are encoded by *ohcBAC* operon, and one or several *ohc* genes are present in other *Desulfovibrio* spp. genomes as well [33]. We observed that a regulatory gene DVU3142 and its orthologs are divergently transcribed from the *ohc* genes in six *Desulfovibrionales* genomes (Fig. 6). A comparative analysis identified σ^54^-dependent promoters upstream of *ohc* genes, thus we predicted that DVU3142 EBP is a transcriptional regulator of Ohc system. We detected DVU3142 binding sites upstream of all *ohc* operons. Remarkably, *D. alaskensis* G20 lacks *ohcA* and *ohcC* genes but the last remaining gene of the Ohc system, *ohcB*, is still regulated by a DVU3142 ortholog since we found σ^54^-dependent promoter and DVU3142 binding site upstream of the *ohcB* gene (Fig. 6).

#### Regulator of alanine dehydrogenase (DVU0569)

L-Alanine dehydrogenase (Ald) catalyzes the oxidative deamination of L-alanine and the reductive amination of pyruvate. Various regulators of alanine dehydrogenase have been characterized in *Bacillus subtilis* (PucR family TF [34]), *Amycolatopsis mediterranei* (GlnR family TF [35]), *Rhizobium leguminozarum* and *Mycobacterium smegmatis* (AsnC family TFs [36, 37]), but no such regulators have been studied in *Deltaproteobacteria*. A gene encoding Ald enzyme in *D. vulgaris* (DVU0571) is located downstream from the regulatory gene DVU0569 as well as in the six other *Desulfovibrionales* genomes. A conserved regulatory motif (Fig. 2) and σ^54^-dependent promoters were found upstream of *ald* genes.

### Evolutionary conservation of σ^54^-dependent regulome in *Desulfovibrionales* species

**Fig. 7:**
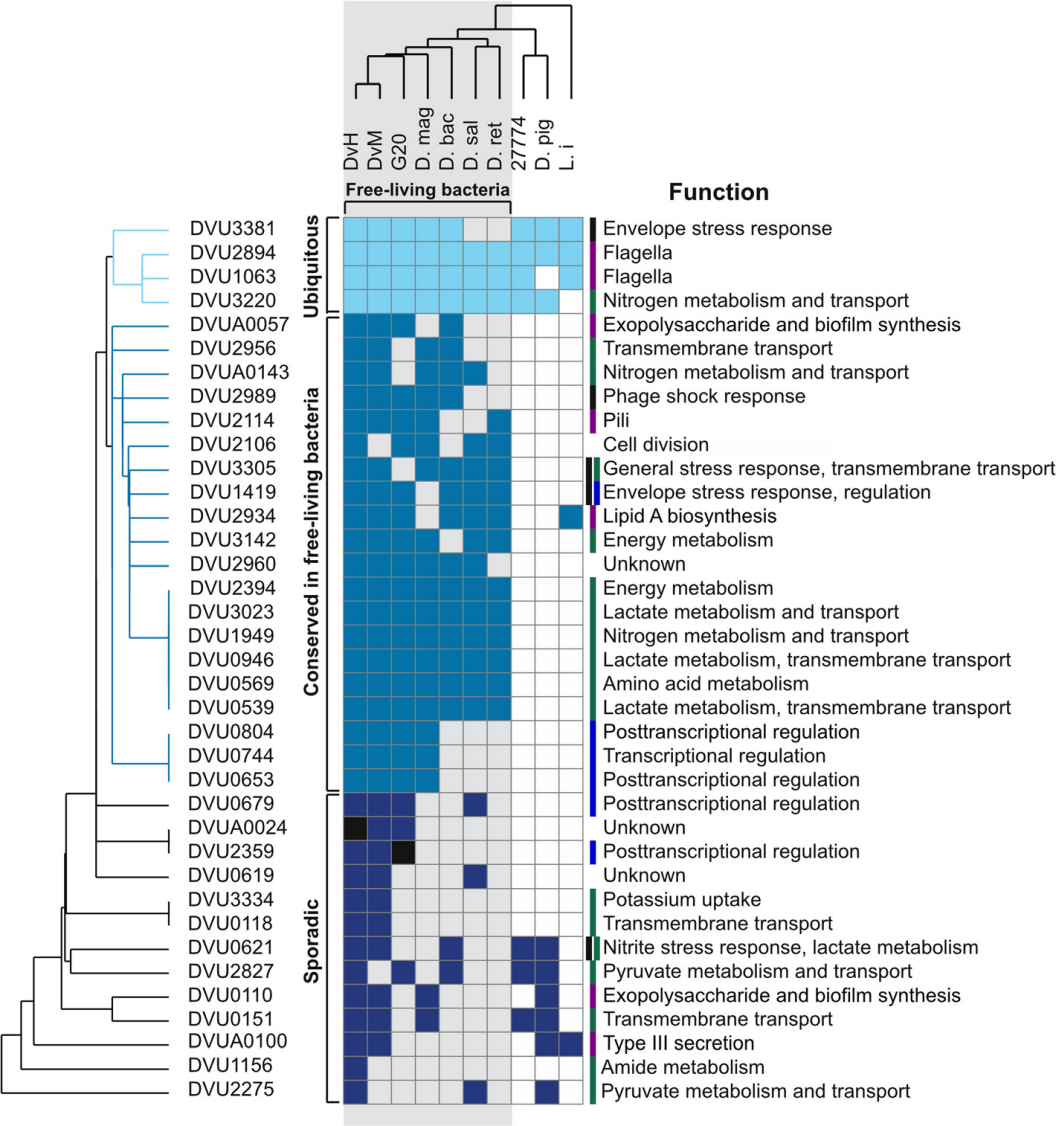
Two-dimensional hierarchical clustering based on conservation of reconstructed σ^54^-dependent regulons across *Desulfovibrionales* genomes. Free-living bacteria are marked with grey background. Pseudogenes of EBPs are marked black. Species abbreviations used: DvH, *Desulfovibrio vulgaris* Hildenborough; DvM: *Desulfovibrio vulgaris* Miyazaki; G20: *Desulfovibrio alaskensis* G20; D. mag: *Desulfovibrio magneticus*; D. bac: *Desulfomicrobium baculatum*; D.sal: *Desulfovibrio salexigens*; D. ret: *Desulfohalobium retbaensae*; 27774: *D. desulfuricans* 27774; D. pig: *Desulfovibrio piger*; L. i: *Lawsonia intracellularis*

To study phylogenetic distribution of the reconstructed σ^54^-dependent regulons, we performed two-dimensional hierarchical clustering based on the presence/absence of reconstructed regulons among *Desulfovibrionales* genomes (Fig. 7). The largest group (20 *D. vulgaris* Hildenborough regulons) is almost exclusively conserved in free-living (i. e. inhabiting such environments as soils, hotsprings, sediments and lakes) bacteria. Many regulons controlling metabolism, extracellular structures, non-coding RNAs and stress response belong to this group. A group of four *D. vulgaris* Hildenborough regulons is conserved in most species, regardless of their lifestyle. This group includes regulons controlling flagellar genes, stress response and nitrogen metabolism. The third group consists of 13 low-conserved regulons that sporadically occur in different genomes.

Clustering results demonstrate that bacterial species are grouped by their lifestyle rather than by their phylogenetic relatedness (see Fig. 3 for comparison). Free-living species are clustered separately from animal host-associated species. The variation in the numbers of σ^54^-dependent regulons among *Desulfovibrionales* species suggest that σ^54^-dependent regulation may play an important role in the adaptation of free-living species to constantly changing environmental conditions. Based on this analysis we speculate that the relative higher abundance of EBPs and associated σ^54^-dependent regulons in free-living *Deltaproteobacteria* is a consequence of their life style. However, neither functional category is strictly limited to free-living bacteria: EBPs regulating metabolism and transport and EBPs regulating extracellular functions may be ubiquitous or be restricted to free-living bacteria. Only regulators of non-coding RNAs were found to have a propensity toward free-living species.

### Co-occurrence of EBP binding sites and σ^54^-dependent promoters in *D. vulgaris* Hildenborough

**Fig. 8:**
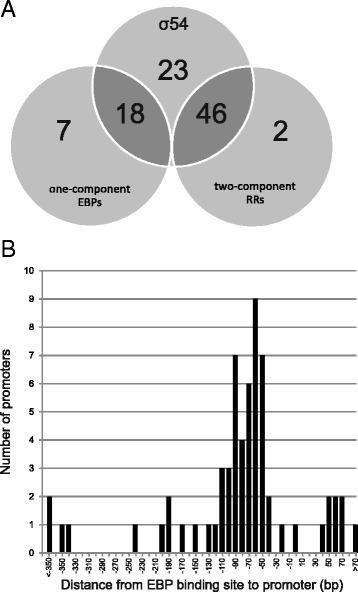
**a** Number of operons in σ^54^-dependent sigmulon, one-component EBP regulome and two-component EBP regulome. Dark grey areas correspond to operons co-regulated by both EBP and σ^54^ protein. Light grey areas correspond to either operons with EBP binding sites or σ^54^-dependent promoters only. **b** Distribution of distances between σ^54^-dependent promoter and closest EBP binding site

Our reconstruction of σ^54^-dependent regulome includes UASs upstream of 64 operons with σ^54^-dependent promoters (Fig. 8a). In addition to these operons, EBP binding sites were found upstream of nine operons that have no σ^54^-dependent promoters. The biggest fraction of σ^54^-dependent promoters were found within 100 nucleotides from the closest EBP binding site (Fig. 8b), while in four cases, the promoter was found at more than 300 bp downstream from the cognate UAS. Further, while EBP binding sites are typically located upstream of the σ^54^-dependent promoter, a small number of UASs were found at a distance of 40–70 bp downstream from the promoters, and most of these were binding sites for the flagellar regulator DVU1063.

For 23 operons with σ^54^-dependent promoters, we could not find predicted UASs (Fig. 8b). These promoters belong to operons from different functional groups, but a majority of them are involved in the composition of the bacterial exterior. In particular, almost half of these operons are predicted to be involved in flagellar biogenesis and motility: five operons encode flagellar components and six operons are co-expressed with flagellar genes (data not shown). The remaining seven promoters without predicted UASs are linked with chemotaxis, exopolysaccharide biosynthesis, biofilm formation, cell wall metabolism, pili and type III secretion. We cannot exclude the possibility that some of these operons may belong to the unidentified DVUA0024 and DVU2960 regulons. However, they also may be regulated by EBP binding sites located far upstream or downstream from the promoter, beyond the limits of our search or within coding regions. For example, an NtrC-dependent enhancer retains its activity even being positioned up to 15 kb away from σ^54^-dependent *glnAp2* promoter of *E. coli* [38]. A high conservation of coding regions precludes us from prediction of such enhancers by computational methods, thus further experimental studies of such cases are needed.

## Conclusions

In this study we comprehensively characterized the genome-wide σ^54^-dependent regulome of *D. vulgaris* Hildenborough. The results include: the σ^54^-dependent EBPs (Table 1), the EBP binding sites (Additional file 4), the σ^54^-dependent promoters (Additional file 6) and the σ^54^-dependent genes and operons (Additional file 5).

Our results demonstrate that the σ^54^-dependent regulome controls a wide variety of cellular functions in *D. vulgaris* Hildenborough. We identified regulatory elements that activate σ^54^-dependent transcription of several dozens of operons. In particular, we inferred several σ^54^-dependent regulons not known before (including those associated with type III secretion, electron transport, pyruvate and formate metabolism). Many of these elements are conserved between *D. vulgaris* Hildenborough and related sulfate-reducing *Deltaproteobacteria* that makes possible the semi-automated inference of σ^54^-dependent regulome in newly sequenced *Deltaproteobacteria* by projection of the collection of σ^54^-dependent regulons reconstructed in this study.

## Methods

### Data sources

In this work we used genome and protein sequences from NCBI GenBank [39] database and MicrobesOnline [10], and the protein domain annotations from MicrobesOnline.

### Identification of enhancer binding proteins (EBPs)

EBPs were identified as proteins with PF00158 domain, AAA+ domain involved in ATP-dependent interaction with σ^54^ protein.

To check for EBP regulatory function, we verified the presence of the peptide ‘GAFTGA’ motif required for the EBP interaction with σ^54^. We used alignments from MicrobesOnline database [10] for comparison with the PF00158 model from PFAM [40].

We identified a set of orthologs of *D.vulgaris* Hildenborough EBPs in other *Desulfovibrionales* genomes using tree-orthologs computed by MicrobesOnline by examining the pre-computed domain-based gene trees [10]. Cluster 3.0 was used for hierarchical two-dimensional clustering [41].

### Identification of σ^54^-dependent promoters

To identify σ^54^-dependent transcripts in *D. vulgaris* Hildenborough genome, we used both the results of experimental analysis and comparative genomics reconstruction. The presence of σ^54^-dependent promoters in the *D. vulgaris* Hildenborough genome have been shown in several studies [15, 42], but the results of computational and experimental analyses were substantially different. A computational analysis [42] identified 70 candidate σ^54^-dependent promoters. A genome-wide experimental analysis of transcription by 5′-RNA-seq [15] identified another set of 70 putative σ^54^-dependent promoters. However, only 19 promoters are present in both lists.

For comparative analysis we constructed a new, taxon-specific motif for recognition of σ^54^-dependent promoters in *Desulfovibrio* spp. The initial motif was deduced from the upstream regions of DVU0316, DVU0318, DVU1032 and DVU1441 genes and their orthologs from five *Desulfovibrio* genomes. These four genes were selected because they are regulated by σ^54^-dependent DVU1063 response regulator [13], and their predicted σ^54^-dependent promoters perfectly fit 5′-RNA-seq data [15]. We used this motif for initial search of σ^54^-dependent promoters in the ten *Desulfovibrionales* genomes using RegPredict [43]. From this search, to refine the σ^54^-dependent promoter motif, we selected those *D. vulgaris* Hildenborough promoters that are located close to the mapped RNA 5′-ends [15] and orthologous promoters from the other nine species. The final motif, based on comparison of the ten genomes, is highly similar to the σ^54^-dependent promoter motif identified by 5′-RNA-seq analysis in *D. vulgaris* Hildenborough [15]. It has two promoter elements similar to −24 and −12 elements from other taxonomic groups [44], with consensus sequences tGGcacg and tTGCt, respectively.

A comparative analysis with the refined σ^54^-dependent promoter motif identified 84 conserved promoters in the *D. vulgaris* Hildenborough genome. Additional three high-score non-conserved σ^54^-dependent promoters were added to our dataset from the 5′-RNA-seq results [15]. Thus, our reconstruction of σ^54^-dependent sigmulon of *D. vulgaris* Hildenborough produced 87 promoters (Additional file 6).

### Reconstruction of regulons for one-component EBPs

To identify binding sites for the one-component σ^54^-dependent regulatory proteins, we applied comparative genomics approach. It assumes that TF binding sites upstream of regulated orthologous genes are conserved in different species [45]. An important requirement is the presence of orthologous TFs in these species. We considered σ^54^-dependent operons co-localized with EBP-encoding genes the most probable targets of these EBPs since bacterial regulatory genes are often co-localized with their regulated genes [46].

We found that ten out of thirteen genes encoding one-component EBPs are co-localized with σ^54^-dependent promoters. Three of these genes, DVU2827, DVU2960 and DVUA0100, are located in σ^54^-dependent operons. Six other genes, DVU0151, DVU2275, DVU2359, DVU2956, DVU2989, DVU3142, are divergently transcribed from σ^54^-dependent promoters. And for one gene encoding EBP, DVU0569, σ^54^-dependent promoter was observed downstream of the gene. In other *Desulfovibrionales* genomes, orthologs of these EBP-encoding genes are co-localized with σ^54^-dependent promoters as well. We used the “Discover profile” tool of the RegPredict web-server [43] for reconstruction of EBP binding motifs. Sets of upstream sequences of orthologous genes (from −650 to +50 with respect to the translation start) were selected and a common motif with highest information content was identified in each set. We identified candidate binding motifs for all ten EBPs upstream of the respective orthologous σ^54^-dependent promoters.

However, for three regulatory genes, DVU1949, DVU2894 and DVUA0143, we could not find conserved σ^54^-dependent promoters nearby and thus used other evidences such as co-expression and genome context analysis as described below.

For DVU2894 gene we found evidence of its co-expression with the genes encoding flagella assembly proteins by analyzing transcriptomics data from MicrobesOnline. In addition, this protein is similar to the flagellar regulators FlrA and FleQ based upon phylogenetic analysis with AAA+ domain-based phylogenetic tree. We proposed that DVU2894 gene is an auto-regulated member of the flagellar regulatory cascade, since it is not regulated by the flagellar regulator DVU1063 [13]. We also found a conserved inverted repeat with consensus yGTCA-N6-TGACr upstream of all DVU2894 orthologs. Since a similar inverted repeat GTCA-N6-TGGC has been observed in the FlrA binding region upstream of *flrBC* operon promoter in *V. cholerae* [47], we predict that this repeat constitutes DVU2894 binding motif.

For the DVUA0143 regulatory gene, genome context analysis did not provide a clue for possible targets, but the following three evidences suggest that DVUA0143 may be a regulator of nitrogenase operon DVUA0015-DVUA0007. First, phylogenetic analysis indicates similarity of its N-terminal domain to AnfA and VnfA, regulators of nitrogenases from *Azotobacter vinelandii* [48]. Second, phylogenetic distribution of the DVUA0143 orthologs in *Desulfovibrionales* corresponds to the distribution of nitrogenase operons: all five of the ten genomes that have *nifHDK* genes also possess DVUA0143 homologs. And, finally, DVUA0143 ortholog is co-localized with nitrogenase operon in *Syntrophobacter fumaroxidans*. Following these conclusions, we found a candidate DVUA00143 binding motif in upstream regions of nitrogenase operons from five *Desulfovibrionales* genomes.

For the gene encoding DVU1949 regulator, genome context analysis indicated a co-transcription with orthologs of DVU1952 gene encoding a hypothetical protein in seven *Desulfovibrionales* genomes. In four genomes, these genes are also co-transcribed with *iorA* and *iorB* genes encoding alpha and beta subunits of indolepyruvate ferredoxin oxidoredctase (an enzyme involved in the metabolism of aromatic amino acids [49]). We proposed that DVU1949 gene is autoregulated and identified a conserved sequence motif upstream of this operon.

The next steps of regulon reconstruction were done with RegPredict, a web-tool for comparative genomic analysis (21). Briefly, EBP binding motifs identified in this work were used for a whole-genome search in upstream regions of coding genes (from −650 to +50 with respect to the translation start) with a threshold equal to a minimal score among all sites reported by the “Discover profile” tool for the motif.

### Refinement of regulon data for two-component σ^54^-dependent regulatory systems

In our previous study [13], we identified target genes of two-component response regulators in *D. vulgaris* Hildenborough by a combination of experimental and computational approaches. In that study we found target genes for 18 of 22 σ^54^-dependent response regulators and assigned TF binding motifs to 13 of them. We could not find TF binding motifs for the rest of TFs since those proteins are conserved in just four or less *Desulfovibrio* genomes available at that time [10]. As more genome sequences of *Desulfovibrionales* spp.*,* both complete and incomplete*,* became available in GenBank [39], we added them to characterize the TF binding motifs. For the incomplete genomes that have orthologs of TF and target genes previously found by DAP-chip, we extracted upstream regions of the respective orthologs for search of conserved sequence motifs. It allowed us to identify TF binding motifs for DVU0110, DVU0621, DVU0653, DVU0744, DVU0804 and DVUA0057 regulatory proteins. For DVU1156 regulator that has no orthologs in *Desulfovibrio* spp., we applied a specific approach for reconstruction of singleton regulons described earlier [50].

Our whole-genome regulon reconstruction with RegPredict demonstrated that TF binding motif predictions fit our existing DAP-chip data [13] and correlate with the predictions of σ^54^-dependent promoters (Additional file 6). However, we could not find TF binding sites upstream of several genes that lack σ^54^-dependent promoters, despite the fact that the binding was demonstrated by DAP-chip (for example, DVU3131 for DVU0804 protein, DVU0736 for DVU0744 protein and DVU0328, DVU0330, DVU1639, DVU2842, DVU1017 for DVUA0057 protein).

For DVU0110 and DVUA0057 EBPs, we predict a common binding motif. Previously, we demonstrated that these two EBPs bind upstream of DVU0132-DVU0133 operon encoding TSUP family permease [13]. Here, we found a conserved motif upstream of this operon in six *Desulfovibrionales* genomes. Two of these genomes have only DVU0110 orthologs (*D. magneticus* and *D. piger*), two genomes have only DVUA0057 orthologs (*D. alaskensis* and *D. baculatum*) and two genomes have both EBPs (*D. vulgaris* strains Hildenborough and Miyazaki). Thus, the found motif cannot be attributed to either of these regulators but only to both of them. Similar binding sites were found upstream of other known DVUA0057 target genes [13] with σ^54^-dependent promoters (DVUA0030, DVUA0032, DVUA0036, DVUA0089), thus suggesting that DVUA0057 binds them.

We also assigned binding sites for DVU0619, a response regulator not studied by DAP-chip in our previous work. Comparative analysis demonstrated that the DVU0617-DVU0616 operon and its homologs in several genomes are co-localized with DVU0619 orthologs, and σ^54^-dependent promoters are present upstream of the operons. A putative DVU0619 binding motif was identified upstream of all these operons.

### Electrophoretic mobility shift assay

For the putative regulatory motif upstream of DVU2957 promoter, we had two neighbor candidate regulatory proteins, DVU2956 and DVU2960. DVU2956 gene is divergently transcribed from the DVU2957-DVU2967 operon, while DVU2960 gene is a part of the operon. The final assignment of a regulatory protein for this motif was done by EMSA. DVU2956 and DVU2960 genes were amplified from *D. vulgaris* Hildenborough genomic DNA and each cloned into vector pSKB3 with a N-terminal His-tag. The resulting constructs were transformed into *E. coli* BL21 (DE3), and His-tagged proteins were expressed and purified as described previously [13]. Full-length EMSA substrate was prepared by amplifying a 370 bp region upstream of DVU2957 with a biotin-labeled reverse primer and an unlabeled forward primer, followed by gel purification. Predicted motif substrates for EMSA were prepared by annealing biotinylated oligonucleotides containing the binding site as described previously [13]. EMSA reactions (20 μl) were set up at room temperature for 20 min with 100 fmol of DNA and purified His-tagged protein (amounts shown in Fig. 4) in 10 mM Tris HCl pH 7.5, 1 mM DTT, 50 mM KCl, 5 mM MgCl_2_ and 1 μg/ml poly dI.dC. Electrophoresis, blotting, and chemiluminescent detection were performed as described previously [13]. Final imaging of the blot was done using the Fluor Chem Q system (Protein Simple, Santa Clara, CA).

## Availability of supporting data

The data sets supporting the results of this article are included within the article and its additional files. The collection of EBP regulons reconstructed with the RegPredict web-server [43] is available in the RegPrecise database, http://regprecise.lbl.gov [51].

## Additional files

Additional file 1:
**EBP taxonomic distribution.** (XLSX 78 kb) Additional file 2:
**EBP DNA-binding domains.** (XLSX 9 kb) Additional file 3: Multiple sequence alignment of homologous regions corresponding to the SSF46785 domain in DVU0151 and its orthologs. (PNG 22 kb) Additional file 4:
**EBP binding sites.** (XLSX 41 kb) Additional file 5:
**σ** ^**54**^ **-dependent genes and operons.** (XLSX 18 kb) Additional file 6:
**σ** ^**54**^ **-dependent promoters.** (XLSX 19 kb)

## Abbreviations

EBP: Enhancer binding protein
MFS: Major facilitator superfamily
OCS: One-component system
PFL: Pyruvate formate-lyase
TF: Transcription factor
TCS: Two-component system
T3SS: Type III secretion system
UAS: Upstream activating sequence
