# Supplementary figures and images for: σ54-dependent regulome in Desulfovibrio vulgaris Hildenborough
Source: BMC Genomics. 2015 Nov 10;16:919. doi: 10.1186/s12864-015-2176-y (PMC4641369; doi:10.1186/s12864-015-2176-y)

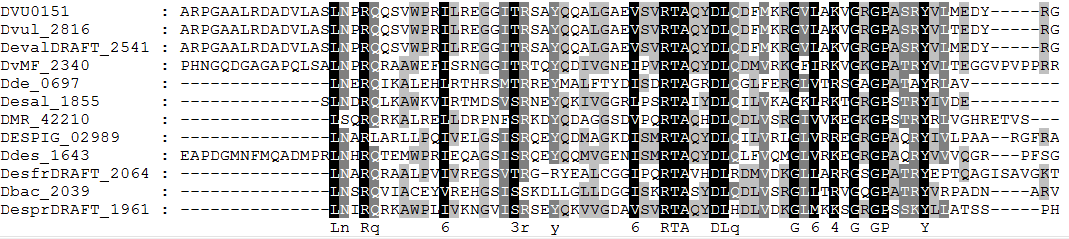

Supplement: Additional file 3: — Multiple sequence alignment of homologous regions corresponding to the SSF46785 domain in DVU0151 and its orthologs. (PNG 22 kb) [file 12864_2015_2176_MOESM3_ESM.png]
